# Mutants of the *white* ABCG Transporter in *Drosophila melanogaster* Have Deficient Olfactory Learning and Cholesterol Homeostasis

**DOI:** 10.3390/ijms222312967

**Published:** 2021-11-30

**Authors:** Jennifer L. Myers, Maria Porter, Nicholas Narwold, Krishna Bhat, Brigitte Dauwalder, Gregg Roman

**Affiliations:** 1 Department of Biology and Biochemistry, University of Houston, Houston, TX 77096, USA; jlmyers21@gmail.com (J.L.M.); bdauwald@central.uh.edu (B.D.); 2 Department of Biology, University of Mississippi, Oxford, MS 38677, USA; mtpena@goolemiss.edu; 3 Department of Molecular Medicine, University of Southern Florida, Tampa, FL 33613, USA; narwold@usf.edu (N.N.); kbhat@usf.edu (K.B.); 4 Department of Biomolecular Sciences, University of Mississippi, Oxford, MS 38677, USA

**Keywords:** olfactory learning, *white*, *Drosophila*, cholesterol, serotonin, dopamine, ABCG transporter, freeze tolerance

## Abstract

*Drosophila*’s *white* gene encodes an ATP-binding cassette G-subfamily (ABCG) half-transporter. White is closely related to mammalian ABCG family members that function in cholesterol efflux. Mutants of *white* have several behavioral phenotypes that are independent of visual defects. This study characterizes a novel defect of *white* mutants in the acquisition of olfactory memory using the aversive olfactory conditioning paradigm. The *w^1118^* mutants learned slower than wildtype controls, yet with additional training, they reached wildtype levels of performance. The *w^1118^* learning phenotype is also found in the *w^apricot^* and *w^coral^* alleles, is dominant, and is rescued by genomic *white* and mini-*white* transgenes. Reducing dietary cholesterol strongly impaired olfactory learning for wildtype controls, while *w^1118^* mutants were resistant to this deficit. The *w^1118^* mutants displayed higher levels of cholesterol and cholesterol esters than wildtype under this low-cholesterol diet. Increasing levels of serotonin, dopamine, or both in the *white* mutants significantly improved *w^1118^* learning. However, serotonin levels were not lower in the heads of the *w^1118^* mutants than in wildtype controls. There were also no significant differences found in synapse numbers within the *w^1118^* brain. We propose that the *w^1118^* learning defect may be due to inefficient biogenic amine signaling brought about by altered cholesterol homeostasis.

## 1. Introduction

*Drosophila*’s *white* gene, necessary for normal eye pigmentation, has one of the longest and most impactful histories of any gene [1,2,3]. Loss-of-function *white* alleles are also commonly found in *Drosophila* experimental genotypes [4,5]. Despite the usefulness of these easily observable genetic markers, the presence of *white* mutant alleles can pose a confounding problem in many experiments because they have pleiotropic behavioral and neural phenotypes, including aspects of learning and memory. Some behaviors are due to poor visual acuity [6,7,8,9], while others appear independent of visual perception [10,11,12,13,14,15,16]. The molecular mechanisms by which the mutant *white* alleles produce these non-visual phenotypes remain mostly uncertain.

One possible explanation for the pleiotropy is the molecular role of white as a broadly selective ATP-binding cassette (ABC) transporter of the G-subfamily [17,18,19]. Many eukaryotic ABC transporters are efflux proteins that transport a broad array of molecules out of the cell and include the breast cancer resistance protein (BCRP, ABCG2) [20,21]. Additionally, some ABC transporters, like white in the pigment granules of the *Drosophila* eye and Malpighian tubules, are involved in transport from the cytoplasm into intracellular compartments [22]. Many ABC transporters are largely involved in the movement of hydrophobic compounds and are associated with metabolism, secretion, and homeostasis [23]. Structurally, most ABC transporters have a minimum of four domains—two nucleotide-binding domains (NBD) and two transmembrane domains (TMD) [20]. Functionally, the TMDs bind substrates and are allosterically coupled to the NBD so that substrate binding enables ATP binding and subsequent ATP hydrolysis to facilitate substrate transport [24,25]. The ABC G-subfamily (ABCG), which includes the white protein, is distinctive because it consists entirely of half transporters that form obligate homo- or heterodimers for function [26,27].

Many ABCG family members have roles in sterol homeostasis [27]. Mammalian ABCG1 and ABCG4 are co-expressed in the brain, most likely as homodimers, and are involved with intracellular sterol transport and regulation of cholesterol homeostasis [27,28,29,30,31]. The ABCG5 and ABCG8 transporters form obligate heterodimers, crucial for the excretion of cholesterols [32,33]. The *Drosophila ATP transporter expressed in trachea* (*Atet*) is located in the fly’s prothoracic gland and functions in exocytotic vesicles as an ecdysone pump [34]. Additionally, the *Drosophila* ABCG gene *CG9663* is transcriptionally upregulated by *Drosophila* hormone receptor 96 (DHR96), a nuclear receptor that responds to changes in cholesterol abundance, suggesting a possible role in cholesterol homeostasis [35]. To our knowledge, the white gene has not been previously examined for a role in cholesterol homeostasis.

The *white* gene has roles in several learning paradigms. Loss-of-function *w^1118^* mutants have severe defects in novelty habituation during exploration, which appear to be primarily due to visual defects [9]. The *w^1118^* mutants have also exhibited attenuated operant place memory when tested in an operant heat box assay [12]. While the *w^1118^* mutants displayed normal levels of short-term olfactory memory in the standard long program paradigm, *w^1118^* short-term memory was higher than wildtype when the electric shock voltage, used as the unconditioned stimulus, was reduced [11]. This enhanced learning found in *w^1118^* is likely due to an increased electric shock sensitivity found in this mutant [11].

Some behavioral differences are hypothesized to result from white’s putative role in serotonin (5-HT) biosynthesis through its participation in tryptophan and guanine transport [12,14]. Consistent with this hypothesis, the defect in operant place memory is phenocopied by inhibition of 5-HT synthesis but not by the inhibition of dopamine biosynthesis [12]. Some measurements from whole fly heads have found a lower level of 5-HT, dopamine, and histamine in *w^1118^* compared to wildtype Canton-S or Oregon-R flies [12,36]. However, the 5-HT levels in wildtype heads from these studies were much higher than previously measured by others [12,36,37,38,39,40]. Moreover, a later study found no difference in 5-HT levels between the dissected brains of *w^1118^* and wildtype Canton-S, suggesting that white may not have a limiting effect in the synthesis of biogenic amines within the central nervous system [40].

In this paper, we demonstrate a role for the *white* gene in olfactory associative learning, and we explore possible mechanisms related to cholesterol homeostasis. We present evidence that suggests *w^1118^* mutants are defective in regulating levels of cholesterol and cholesterol esters. Furthermore, increasing either dopamine or 5-HT levels in the *w^1118^* mutants partially rescues the learning defect. Yet, we found that 5-HT levels in the *w^1118^* heads were somewhat higher than those of our wildtype control flies. We propose a hypothesis whereby defects in cholesterol homeostasis in the *w^1118^* mutants lead to less effective signaling through 5-HT and dopamine receptors and, consequently, account for less efficient acquisition of learning.

## 2. Results

### 2.1. Mutants of white Have Poor Learning Acquisition

In the *Drosophila* “long program” olfactory learning paradigm, 12 electric shocks are delivered during a one-minute presentation of the odorant-paired conditioning stimulus (CS+) [41,42]. Each electric shock represents a single training trial, and an acquisition curve can be generated by varying the number of shocks paired with the CS+ odorant. The 12 shocks presented in the long program saturate the amount of learning in wildtype lines [41,43]. Acquisition curves can be used to identify mutants that have defects in their rate of learning, while maintaining wildtype memory formation or recall.

**Figure 1:**
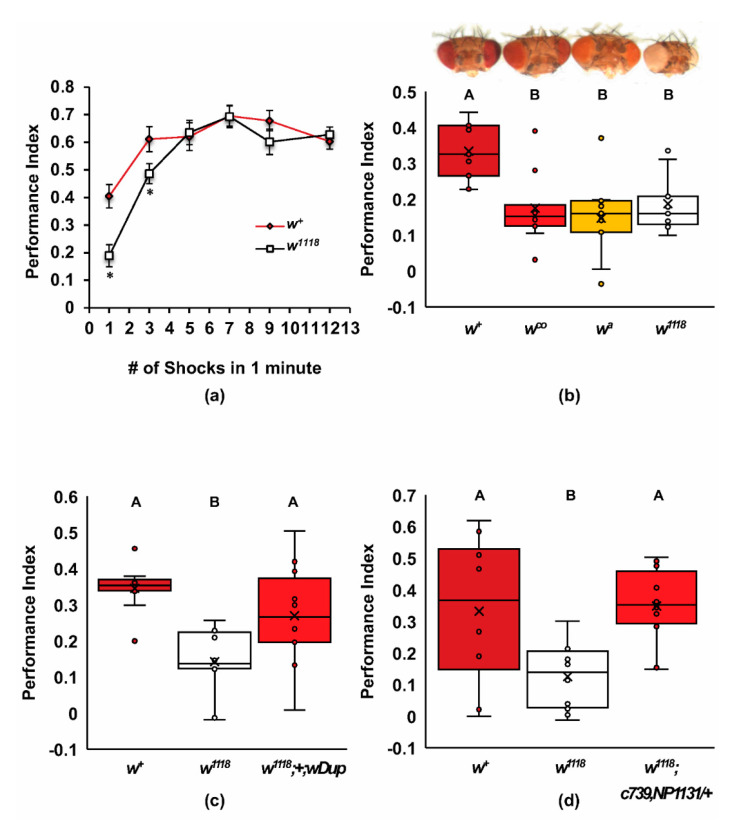
Mutants of *white* have a defect in acquisition that can be rescued by *white*^+^ and mini-*white* transgenes. (**a**) Olfactory learning acquisition curves are shown for *w^1118^* mutants and wildtype Canton-S (*w^+^*). The *w^1118^* mutant has lower associative STM with one and three shock pairings to odor (two-way ANOVA, Adj. *R*^2^ = 0.475, *F*_6,121_ = 20.157, *p* < 0.0001, *n* = 10–13; Tukey post hoc *w^+^* versus *w^1118^*, *p* < 0.05, *n* = 64); (**b**) one-trial olfactory learning for wildtype and the *w^coral^*, *w^apricot^*, and *w^1118^* mutants are shown. Representative photos of the heads are provided above the graph to demonstrate the level of *white* activity remaining in eye pigment cells for each mutant. All mutants were equally poor in associative STM and significantly different from wildtype (ANOVA, Adj. *R*^2^ = 0.321, *F*_3,32_ = 6.524, *p* < 0.001, *n* = 9); (**c**) poor one-trial learning for *w^1118^* was rescued by a genomic duplication (*w^1118^*; Dp(1;3)DC050- wDup; ANOVA, Adj. *R^2^* = 0.356, *F*_2,27_ = 9.022, *p* < 0.001; Tukey post hoc, *p* < 0.05; *n* = 10); (**d**) poor one-trial learning for *w^1118^* was rescued by two mini-*white* marked GAL4 drivers (ANOVA, Adj. *R*^2^ = 0.254, *F*_2,25_ = 5.590, *p* < 0.01; Tukey post hoc, *p* < 0.05; *n* = 8–10). In panels b, c, and d, groups that do not share letters above the box plots are significantly different from each other according to the Tukey post hoc test (* *p* < 0.05).

The *w^1118^* mutants used in this study were previously shown to have normal levels of learning in the long program [11,41]. We found that the *w^1118^* loss-of-function mutants had defects in acquiring olfactory associative memory and that these defects were overcome by additional training trials (Figure 1a). These mutants had significantly lower performance indices at one and three training trials, but by five training trials had caught up to the performance of the wildtype Canton-S controls (two-way ANOVA, Adj. *R*^2^ = 0.475, *F*_6,121_ = 20.157, *p* < 0.0001, *n* = 10–13; Tukey post hoc *w^+^* versus *w^1118^*, *p* < 0.05, *n* = 64). We did not find any significant differences between naïve responses of *w^1118^* and wildtype flies and the olfactory or electric shock controls (Supplementary Table S1). These data indicate a slower rate of learning phenotype for the *w^1118^* mutants.

To verify that the learning defect in *w^1118^* was due to a deficiency in *white* gene activity, we examined independent alleles and transgenic rescue constructs using the one-trial learning protocol (Figure 1b–d). The hypomorphic *white^apricot^ (w^a^)* and *white^coral^ (w^co^*) mutants also had significantly lower performance indices after one-trial learning, comparable to the amorphic *w^1118^* mutants (Figure 1b; ANOVA, Adj. *R*^2^ = 0.321, *F*_3,32_ = 6.524, *p* < 0.001, *n* = 9). The decrease in performance for all three *w* mutants was about 50%, regardless of the strength of the eye phenotype, suggesting that olfactory learning is very sensitive to reduced levels of *white* activity. The *w^1118^* one-trial learning phenotype was rescued by including a genomic duplication of the *w* locus (Figure 1c; ANOVA, Adj. *R*^2^ = 0.356, *F*_2,27_ = 9.022, *p* < 0.001; Tukey post hoc, *p* < 0.05; *n* = 10). This duplication was a 93,901 base pair genomic fragment of the X chromosome, containing the entire *white* gene near its center, and also included the *CG12498*, *CG32795*, and the *IncRNA:CR43494* genes; this duplication was docked on the left arm of chromosome 3 [44]. We further found that learning was restored in *w^1118^* flies by the addition of two copies of mini-*white* contained within two separate GAL4 driver lines, c739 and NP1131 (Figure 1d, ANOVA, Adj. *R*^2^ = 0.254, *F*_2,25_ = 5.590, *p* < 0.01; Tukey post hoc, *p* < 0.05; *n* = 8–10). The mini-*white* construct used in these GAL4 drivers contains an hsp70 promoter that drives relatively high levels of mini-*white* gene expression [45]. These allele and rescue data indicate that relatively high levels of *white* activity are required for wildtype rates of olfactory learning. 

### 2.2. Screen for Additional ABCG Learning Mutants

**Figure 2:**
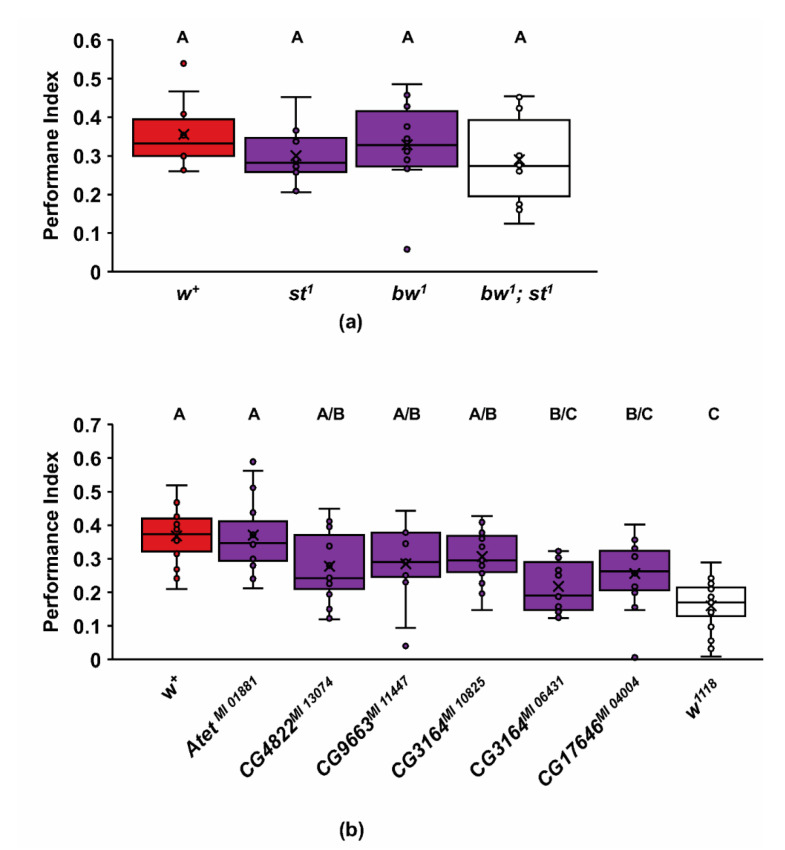
Assessment of one-trial learning in *Drosophila* ABCG mutants. (**a**) Outcrossed mutants of ABCG transporters *scarlet* (*st^1^*), *brown* (*bw^1^*), and *bw^1^; st^1^* were not statistically different from wildtype (*w^+^*) (ANOVA, Adj *R*^2^ = −0.015, *F*_3,36_ = 0.809, *p* = 0.497; Tukey post hoc, *p* < 0.05; *n* = 10); (**b**) the *Atet^MI 01881^*, *CG4822^MI 13074^*, *CG9663^MI 11447^*, *CG3164^MI 10825^*, *CG3164^MI 06431^*, and *CG17646^MI 04004^* homozygous mutants were compared to both *w^+^* flies and *w^1118^* mutants. All ABCGs except *Atet^MI 01881^* had lower acquisition performance than *w^+^*. *CG3164^MI 06431^* and *CG17646^MI 04004^* were both significantly different from *w^+^* and not different from *w^1118^* after Tukey post hoc analysis. (ANOVA, Adj. *R*^2^ = 0.309, *F*_7,114_ = 8.717, *p* < 0.0001; Tukey post hoc, *p* < 0.05; *n* = 15). Groups that do not share letters above the box plots are significantly different from each other according to the Tukey post hoc test (*p* < 0.05).

White can homodimerize or heterodimerize with other ABCG half-transporters for normal functions. For example, white is known to heterodimerize with both brown and scarlet ABCG transporters to produce the drosopterin and ommochrome screening pigments, respectively, within the eye’s pigment cells [46,47]. However, mutants of *scarlet^1^* (*st^1^*), *brown^1^* (*bw^1^*), and the *bw^1^*; *st^1^* double mutant all show levels of learning comparable to wildtype Canton-S flies (Figure 2a; ANOVA, Adj *R*^2^ = −0.015, *F*_3,36_ = 0.809, *p* = 0.497; Tukey post hoc, *p* < 0.05; *n* = 10). The *bw^1^* and *st^1^* are both loss of function mutations, and the *bw^1^*; *st^1^* double mutants are white-eyed, lacking all ommochromes and drosopterins [48,49]. This double mutant’s failure to mimic the *w^1118^* olfactory learning phenotype suggests that white’s function in the eye is, not surprisingly, independent of its role in learning. Moreover, the transport functions of the white–brown and white–scarlet transporters are not required for olfactory learning.

In search of other potential heterodimerization partners, we examined five additional ABCGs that are expressed in *Drosophila* adult heads [50] and are highly homologous to white for defects in one-trial olfactory learning (Figure 2b) [18]. To accomplish this, we examined homozygotes for Mi{MIC} element insertions within these genes, all predicted to severely disrupt activity [51]. Although several of the ABCG mutants we tested appeared to trend lower than wildtype Canton-S, only the *CG17646^MI 04004^* and *CG3164^MI 06431^* mutants were significantly different from wildtype, while not different from *w^1118^* (Figure 2b; ANOVA, Adj. *R*^2^ = 0.309, *F*_7,114_ = 8.717, *p* < 0.0001; Tukey post hoc, *p* < 0.05; *n* = 15). All ABCGs except *w^1118^* had lower naïve odor avoidance for MCH and/or 3-OCT (Supplementary Table S1). In contrast, all the ABCG mutants had normal shock avoidance (Supplementary Table S1). The *Atet^MI 01881^* mutant showed robust learning levels despite low odor avoidance. This result suggests that the odor detection threshold required for maximal olfactory learning may be lower than the threshold for naïve avoidance. Nevertheless, we cannot rule out at this time that the learning defects seen in the *CG3164^MI 06431^* and *CG17646^MI 04004^* mutants are possibly due to defects in olfactory sensitivity. 

**Figure 3:**
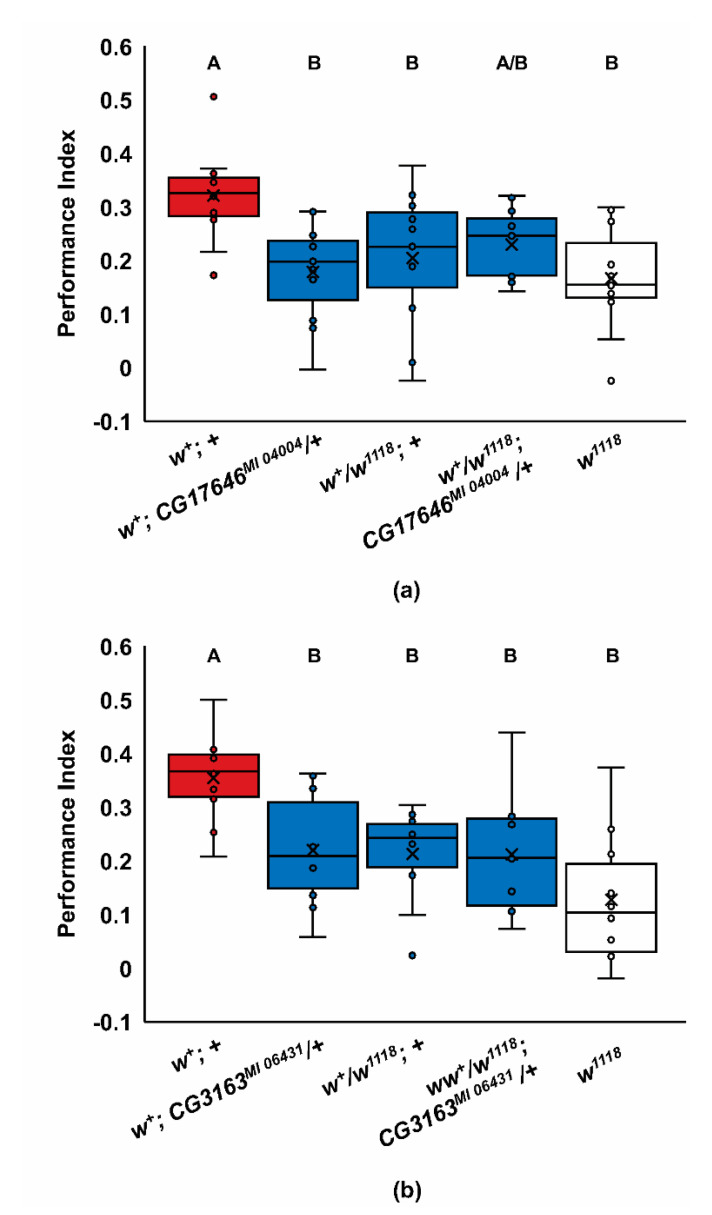
Reduced one-trial learning for *Drosophila* ABCG mutants is not additive with *w^1118^*. (**a**) Crosses were made to produce *CG17646^MI 04004^* heterozygotes, *w^1118^* heterozygotes, or transheterozygotes of *w^1118^* and *CG17646^MI 04004^*. The *w^1118^* and *CG17646^MI 04004^* alleles have dominant learning phenotypes and are not additive for reduced acquisition (ANOVA, Adj. *R*^2^ = 0.205, *F*_4,50_ = 4.485, *p* < 0.004; Tukey post hoc, *p* < 0.05; *n* = 11); (**b**) crosses were made to produce *CG3164^MI 064314^* heterozygotes, *w^1118^* heterozygotes, or trans-heterozygotes of *w^1118^* and *CG3164^MI 06431^*. The *w^1118^* and *CG3164^MI 06431^* alleles have dominant learning phenotypes and are not additive for reduced acquisition (ANOVA, Adj. *R*^2^ = 0.299, *F*_4,45_ = 6.227, *p* < 0.001; Tukey post hoc, *p* < 0.05; *n* = 10). In panels a and b, groups that do not share letters above the box plots are significantly different from each other according to the Tukey post hoc test (*p* < 0.05).

We further examined the learning phenotypes of the *CG17646^MI 04004^* and *CG3164^MI 06431^* mutants by asking if the poor learning phenotypes of these ABCG mutants and *w^1118^* were dominant and if there were phenotypic interactions with *w^1118^* in learning (Figure 3). Since *white* is on the X chromosome, we used only females in these experiments. There were significant effects of genotype in this experiment (Figure 3a; ANOVA, Adj. *R*^2^ = 0.205, *F*_4,50_ = 4.485, *p* < 0.004; Tukey post hoc, *p* < 0.05; *n* = 11; Figure 3b; ANOVA, Adj. *R*^2^ = 0.299, *F*_4,45_ = 6.227, *p* < 0.001; Tukey post hoc, *p* < 0.05; *n* = 10). Interestingly, the *w^1118^* learning phenotype was completely dominant for one-trial learning (Figure 3a,b; *p* < 0.05). This dominance, together with the *w^co^* and *w^a^* phenotypes, indicates that learning is highly sensitive to *white* activity and that the wildtype phenotype requires high levels of *white* expression. In addition, since the *w^+^/w^1118^* heterozygotes were generated from wildtype female virgins, this indicates that the learning phenotype is not maternally inherited. Similar to *w^1118^*, both the *CG3164^MI 06431^* and *CG17646^MI 04004^* mutant alleles also had dominant learning phenotypes (Figure 3a,b). The *CG17646^MI 04004^*/+ heterozygotes had mostly wildtype odor avoidance, suggesting the mutant avoidance phenotype is recessive, or perhaps semi-dominant (Supplementary Table S1). In contrast, the *CG3164^MI 06431^*/+ heterozygotes’ odor avoidance defects were fully dominant (Supplementary Table S1). We failed to find any additive genetic interactions between *w^1118^* and the *CG17646^MI 04004^* or *CG3164^MI 06431^* alleles in the respective transheterozygotes, consistent with these genes affecting the same learning processes (Figure 3); however, given the low level of learning in these genotypes, floor effects may have limited our ability to detect additive interactions. Together these data suggest the possibility that white may heterodimerize with either CG17646*^MI 04004^* or CG3164*^MI 06431^*, or both, to support learning. 

### 2.3. A Role for white in Cholesterol Homeostasis

A potential mechanism for white’s role in learning, and possibly also for either CG17646 or CG3164, is suggested by the roles of the mammalian ABCG1, ABCG4, ABCG5, and ABCG8 transporters in cellular cholesterol efflux [32,33,52]. Unlike mammals that are capable of de novo cholesterol synthesis, *Drosophila melanogaster* is a cholesterol auxotroph [53]. If white participates in the efflux of sterols, reducing the levels of *white* should affect cholesterol abundance and availability within the nervous system. Even subtle changes in cholesterol levels could affect presynaptic vesicle release and recycling, and postsynaptic receptor trafficking and localization in the plasma membrane in a way that reduces the rate of olfactory learning [54,55,56]. 

**Figure 4:**
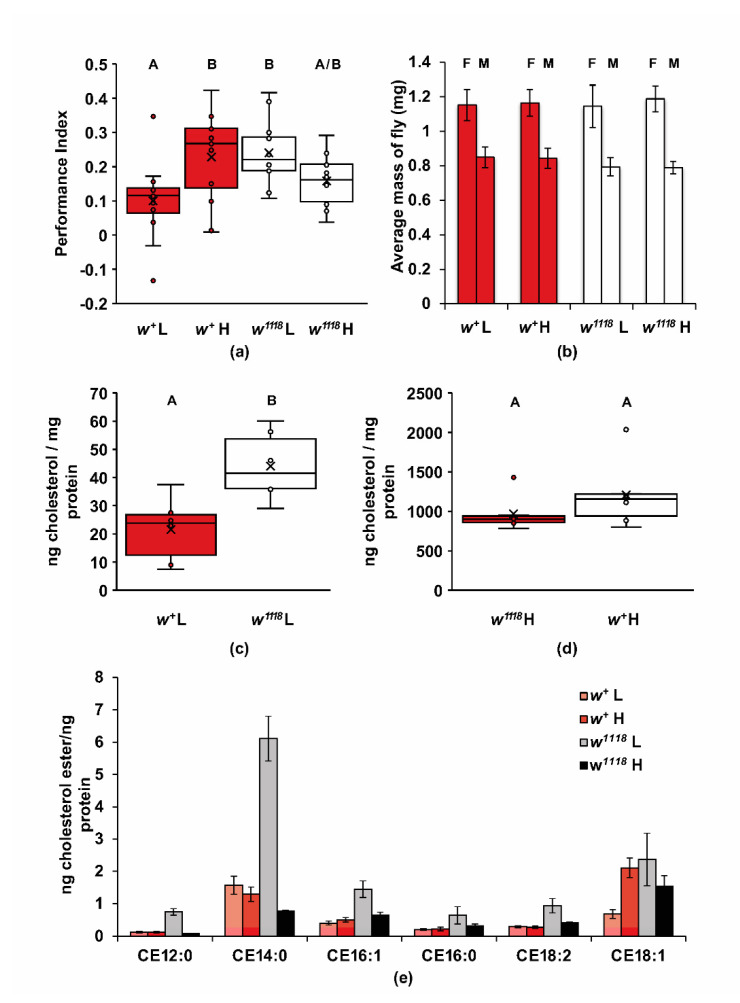
The *w^1118^* mutants have differential responses to dietary cholesterol in one-trial learning and in cholesterol accumulation in their corpses. Canton-S (*w^+^*) and *w^1118^* flies were reared on a low- or high-cholesterol (0.1 mg/mL added) diet. (**a**) Dietary cholesterol had a significant effect on one-trial learning (two-way ANOVA, Adj. *R*^2^ = 0.183, *F*_3,44_ = 4.508, *p* < 0.008; Tukey post hoc, *p* < 0.05; *n* = 12). Lower dietary cholesterol led to impaired learning in wildtype flies (*p* < 0.05), but not in *w^1118^* mutants (*p* = 0.249); (**b**) fly mass was unaffected by genotype or dietary cholesterol for both males and females (two-way ANOVA, Adj. *R*^2^ = −0.138, *F*_3,19_ = 0.111, *p* = 0.952, *n* = 5–6); (**c**) the cholesterol levels in the *w^1118^* mutants were significantly higher than wildtype flies grown when both were raised on the low-cholesterol diet (*t*(10) = 3.292, *p* < 0.01, *n* = 6); (**d**) The cholesterol levels in *w^1118^* mutants were not significantly higher than wildtype flies grown when both were raised on the high-cholesterol diet (*t*(10) = 1.185, *p* = 0.263, *n* = 6); (**e**) *w^1118^* mutants raised on the low-cholesterol diet have greatly increased cholesterol esters compared to wildtype controls and *w^1118^* mutants raised on the high-cholesterol diet (*n* = 3).

To test this possibility, we first asked if olfactory learning is sensitive to levels of dietary cholesterol (Figure 4a). Wildtype and *w^1118^* flies were grown from embryos on a minimal diet of sucrose, yeast, and agar, with or without 0.1 mg/mL of cholesterol. Wildtype flies raised on a low-cholesterol diet had significantly lower performance in one-trial learning than the flies raised on a high-cholesterol diet, indicating a wildtype need of dietary cholesterol for olfactory learning (two-way ANOVA, Adj. *R*^2^ = 0.183, *F*_3,44_ = 4.508, *p* < 0.008; Tukey post hoc, *p* < 0.05; *n* = 12). The *w^1118^* mutant flies performed significantly better than wildtype flies on the low-cholesterol diet, in contrast to their performance on normal food (Tukey post hoc, *p* < 0.05). Hence, the *w^1118^* mutation leads to a resistance to the effect of low dietary cholesterol on learning. Increasing dietary cholesterol in *w^1118^* flies did not lead to further increases in learning in *w^1118^* flies as it did in wildtype flies (Figure 4a; *p* = 0.249). It is interesting that the slow learning phenotype of *w^1118^* mutants was not present under these sterol restrictive diets. These results suggest that cholesterol homeostasis is altered in *w^1118^* mutants in a manner that changes the optimal level of dietary cholesterol for learning and perhaps other cholesterol-dependent phenotypes.

To test if differences in cholesterol homeostasis could account for the *w^1118^* learning phenotype, we compared cholesterol and cholesterol ester levels in *w^1118^* mutants and wildtype control flies fed either a low- or high-cholesterol diet (Figure 4). The dietary cholesterol changes in these experiments did not affect the mass of either wildtype or *w^1118^* mutants (Figure 4b; two-way ANOVA, Adj. *R*^2^ = −0.138, *F*_3,19_ = 0.111, *p* = 0.952, *n* = 5–6). To analyze the uptake and metabolism of dietary cholesterol under each condition, the flies were raised on the same diets used in the previous learning experiment, and then levels of cholesterol and cholesterol esters from frozen whole flies were measured by LC-MS. On the low-cholesterol diet, the *w^1118^* mutants had approximately twice the level of cholesterol compared to the wildtype control (Figure 4c; *t*(10) = 3.292, *p* < 0.01, *n* = 6). On the high-cholesterol diet, the levels of cholesterol extracted from the corpses of both genotypes were approximately 50-fold higher and with greater variability as compared to the low-cholesterol diet; the differences in cholesterol levels between the *w^1118^* and wildtype flies on this high-cholesterol diet were, however, not significant (Figure 4d; *t*(10) = 1.185, *p* = 0.263, *n* = 6). 

Since cellular cholesterol is esterified for storage and transport [57], we also measured the levels of cholesterol esters in the corpses of *w^1118^* and control flies on low- and high-cholesterol diets. There were increases in several individual cholesterol ester levels of *w^1118^* flies on the low-cholesterol diet compared to all other groups (Figure 4e; *n* = 3). While the low sample number makes significance testing less reliable, the effect of a low-cholesterol diet on elevation of cholesterol esters for *w^1118^* flies was consistent and most noticeable for CE 14:0 (cholesterol myristate) in Figure 4e. The effect of diet on cholesterol ester levels of wildtype flies was not appreciatively different except for CE18:1 (Figure 4e). There were also no significant differences in total cholesterol esters between the *w^1118^* mutants grown on the high-cholesterol diet and either wildtype group (Figure S1; Tukey, *p* < 0.05). However, there was a strong increase in the total cholesterol esters of *w^1118^* flies fed a low-cholesterol diet (Figure S1; Tukey, *p* < 0.05). These differences in total cholesterol and cholesterol ester levels and response to dietary cholesterol levels in *w^1118^* mutants are consistent with a role for white in regulating cholesterol homeostasis. 

**Figure 5:**
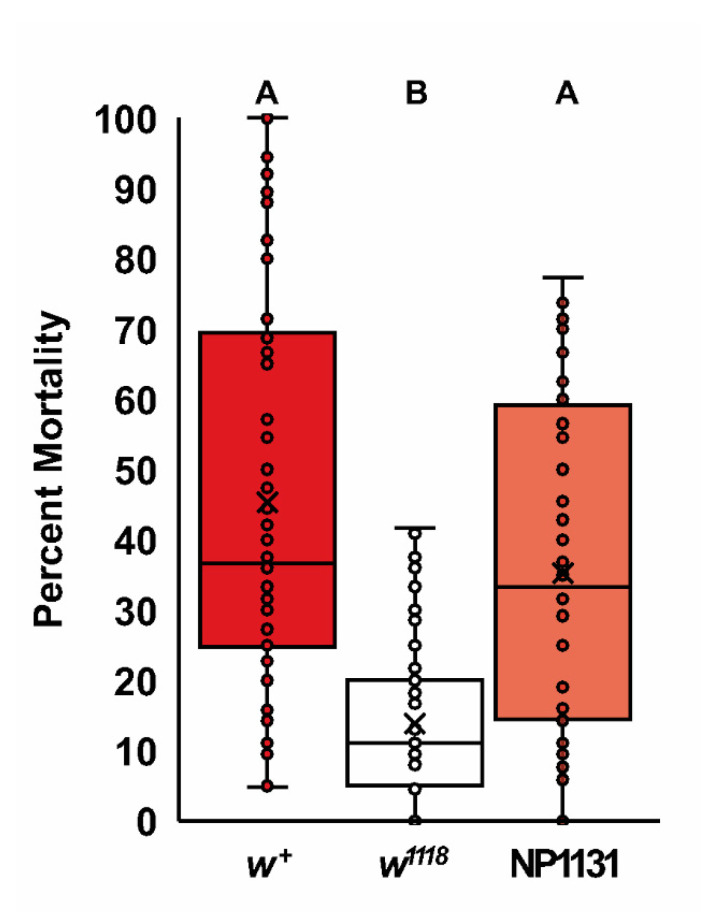
The *w^1118^* mutants have increased freeze tolerance. Wildtype Canton-S (*w^+^*), *w^1118^*, and *w^1118^*; NP1131(mini-*white*) genotypes were subjected to 120 min at −5 °C, followed by a 24 h recovery at 25 °C. The median percentage of mortality after this recover period is shown as the center line, with the mean indicated by the x. There were significant differences in mortality between genotypes (Kruskal–Wallis, *k* = 73.404, *p* < 0.0001, *n* = 64–68). The *w^1118^* flies survived the subfreezing temperature significantly better than *w^+^* and *w^1118^*; NP1131 (Dunn’s procedure, *p* < 0.0001; *n* = 64–68). Groups that do not share letters above the box plots are significantly different from each other according to the Dunn’s post hoc test (*p* < 0.05).

Cellular injury from subfreezing temperatures is primarily due to membrane damage [58]. The susceptibility to this membrane damage can be partially ameliorated by increasing membrane fluidity through changes in membrane lipid composition, including through higher cholesterol levels [59,60]. Cholesterol levels affect freeze tolerance in *Drosophila*, where flies with higher levels of cholesterol can better survive subfreezing temperatures [59]. Since *w^1118^* mutants have altered cholesterol homeostasis, we asked if they also have an altered freeze tolerance. In this experiment, we examined the ability of control flies, *w^1118^* mutants, and *w^1118^* mutants containing a mini-*white* transgene (NP1131) to survive a two-hour incubation at −5 °C (Figure 5). There was a significant effect of genotype in this experiment (Kruskal–Wallis, *k* = 73.404, *p* < 0.0001, *n* = 64–68). The *w^1118^* mutants displayed greatly reduced mortality after freezing compared to wildtype flies (Dunn’s procedure, *p* < 0.0001), and this *w^1118^* freeze-tolerance phenotype was reversed by the presence of a mini-*white* transgene (Dunn’s procedure, *p* < 0.0001), suggesting the increased tolerance is due to a reduction in white activity. This change in freeze tolerance is consistent with *w^1118^* mutants having increased membrane fluidity through higher levels of cholesterol and/or other lipids. 

### 2.4. Biogenic Amine Signaling and the w^1118^ Learning Phenotype

Another possible mechanism for the role of white in learning is through differences in biogenic amine signaling. Serotonin (5-HT) and dopamine levels were previously reported to be reduced in *w^1118^*, *bw^1^*, and *st^1^* mutants [12,36]. However, a subsequent study has failed to find a difference between *w^1118^* and the wildtype in biogenic amine levels using a different detection method [40]. Considering both 5-HT and dopamine function in olfactory learning and memory, reducing either the levels of these amines or reducing the efficacy of their signaling pathways could impact the synaptic processes involved in forming olfactory associative memories [61,62,63]. 

**Figure 6:**
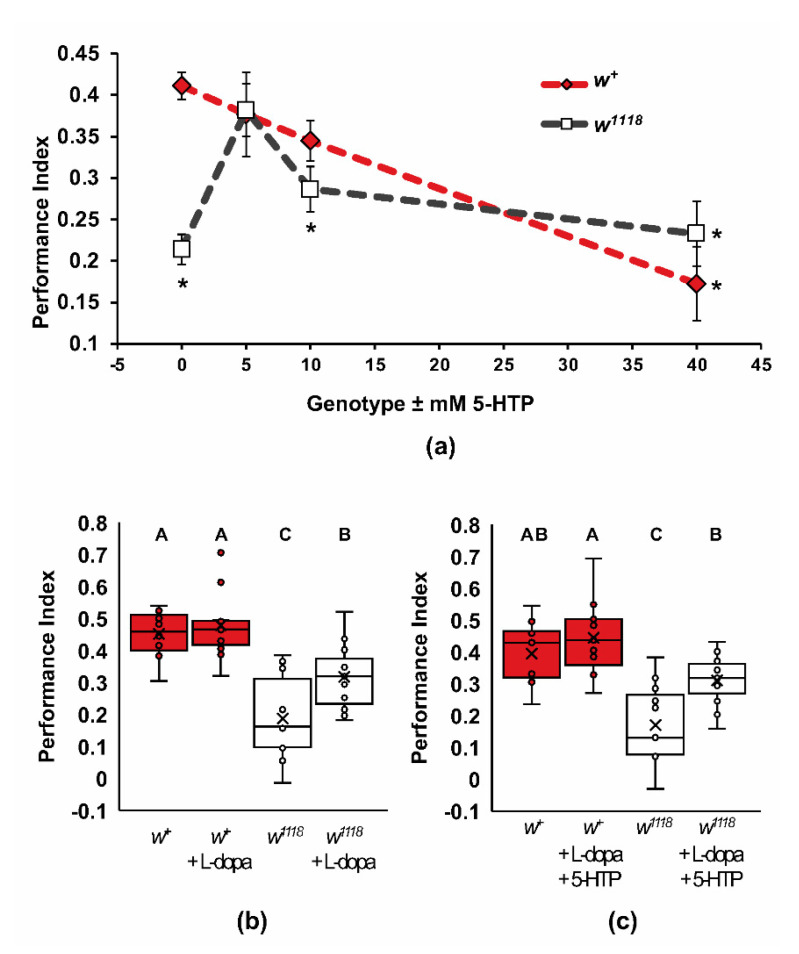
5-HTP and L-dopa partially rescue wildtype acquisition for *w^1118^* mutants. (**a**) Canton-S (*w^+^*) and *w^1118^* flies were fed 5-HTP or vehicle and then examined for one-trial learning. Vehicle-fed flies for all three experiments were averaged for 0 mM, *n* = 37. Compared to the optimal acquisition of wildtype controls, 0mM, 10 mM, and 40 mM 5-HTP-fed *w^1118^*, and 40 mM 5-HTP-fed wildtype flies showed reduced learning (two-way ANOVA, Adj. *R*^2^ = 0.348, *F*_7,140_ = 12.208, *p* < 0.0001, Dunnett post hoc for *w^+^* 0 mM 5-HTP as the control group for comparison, * *p* < 0.01). We found that 5 mM of 5-HTP improved *w^1118^* acquisition compared to untreated *w^1118^*, while 10 mM and 40 mM 5-HTP-fed *w^1118^* flies retained low acquisition (Tukey post hoc, *p* < 0.05); (**b**) Canton-S (*w^+^*) and *w^1118^* flies were fed L-dopa or vehicle and examined for one-trial learning. L-dopa partially rescued wildtype acquisition for *w^1118^* mutants. (two-way ANOVA, Adj. *R*^2^ = 0.522, *F*_3,39_ = 16.308, *p* < 0.0001; Tukey post hoc, *p* < 0.05, *n* = 10–11); (**c**) feeding both 5-HT and L-dopa partially rescued the *w^1118^* one-trial learning phenotype (two-way ANOVA, Adj. *R*^2^ = 0.467, *F*_3,40_ = 13.544, *p* < 0.0001; Tukey post hoc, *p* < 0.05, *n* = 11). In panels b and c, groups that do not share letters above the box plots are significantly different from each other according to the Tukey post hoc, test (*p* < 0.05).

To test the hypothesis that reduced biogenic amine signaling in *w^1118^* mutants causes the learning defect, we examined the impact of artificially raising the levels of 5-HT and dopamine through dietary supplementation (Figure 6). Wildtype and *w^1118^* flies were fed 0, 5, 10, and 40 mM 5-HTP, the metabolic precursor to 5-HT (Figure 6a). These concentrations of 5-HTP, when fed to adult *Drosophila*, raise the levels of 5-HT extracted from heads [64]. In wildtype flies, increasing the levels of 5-HT led to a dose-dependent decrease in one-trial olfactory learning, with significantly lower performance found at 40 mM 5-HTP (two-way ANOVA, Adj. *R*^2^ = 0.348, *F*_7,140_ = 12.208, *p* < 0.0001, Dunnett post hoc, * *p* < 0.01). The *w^1118^* mutants had a more complex response to increasing levels of 5-HT. A slight increase in 5-HT levels, induced by 5 mM 5-HTP feeding, resulted in a rescue of the learning phenotype (Figure 6a; Dunnett post hoc, *p* < 0.01). At 10 mM and 40 mM 5-HTP, the performance of *w^1118^* was significantly lower than the untreated wildtype control (Dunnett post hoc, *p* < 0.01) and not significantly different from each other or untreated *w^1118^* (Tukey, *p* < 0.05), supporting the previous findings by Yarali, et al. [40], who found no difference in either dopamine or 5-HT levels in *w^1118^* mutants. These data suggest that 5-HT signaling in the *w^1118^* mutants is insufficient to support wildtype rates of learning. 

We also asked if raising dopamine levels in the *w^1118^* mutants could impact learning. Feeding flies 2 mg/mL of the dopamine precursor L-dopa increases dopamine to levels that are sufficient for different behavioral responses [8,65]. This treatment resulted in a partial but significant increase in learning in the *w^1118^* mutants, but it did not affect wildtype learning (Figure 6b; two-way ANOVA, Adj. *R*^2^ = 0.522, *F*_3,39_ = 16.308, *p* < 0.0001; Tukey post hoc, *p* < 0.05, *n* = 10–11). These data suggest that dopamine signaling in *w^1118^* is also insufficient to achieve wildtype rates of learning. We further asked if there is a significant interaction between increasing 5-HT and L-dopa in the *w^1118^* mutants (Figure 6c). Feeding wildtype flies both 5mM 5-HTP and 2 mg/mL L-dopa did not significantly affect learning (two-way ANOVA, Adj. *R*^2^ = 0.467, *F*_3,40_ = 13.544, *p* < 0.0001; Tukey post hoc, *p* < 0.05, *n* = 11); however, the *w^1118^* mutants achieved a partial rescue of learning, like that of L-dopa alone (*p* < 0.05).

**Figure 7:**
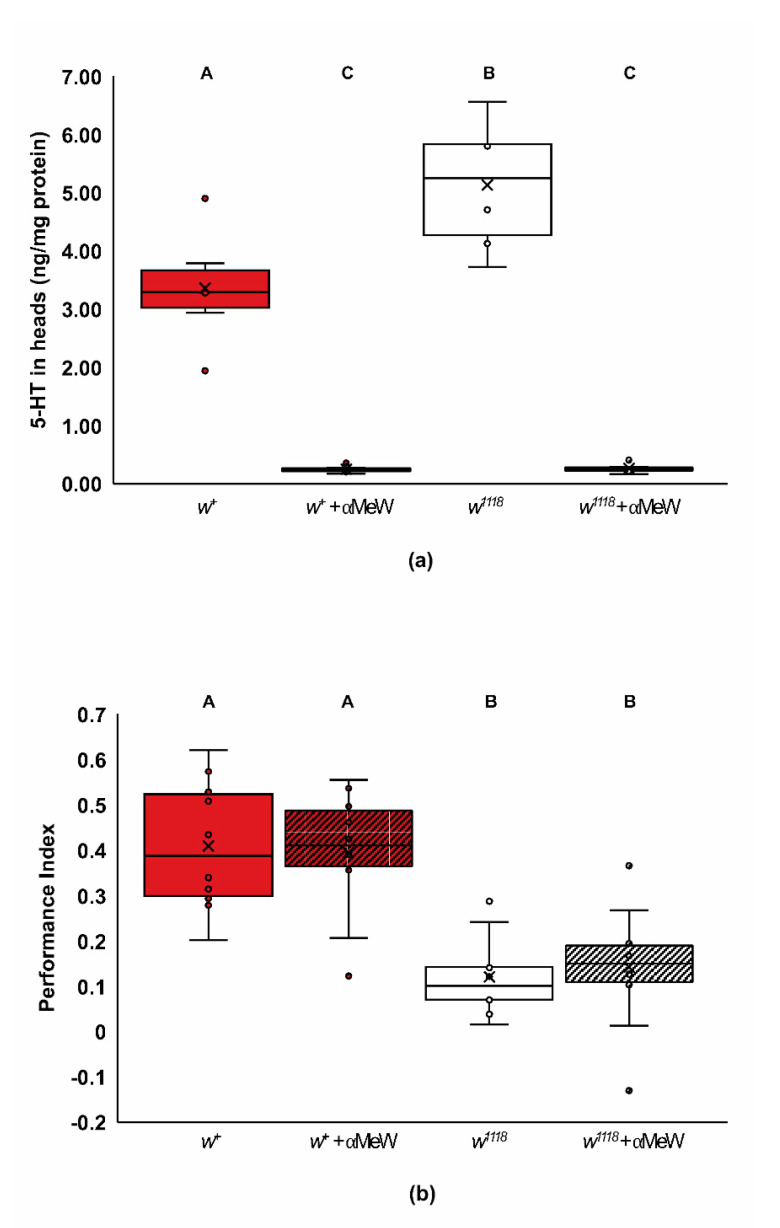
*w^1118^* mutants have increased levels of 5-HT within their heads. (**a**) 5-HT levels normalized against extracted protein concentrations are shown. Wildtype Canton-S (*w^+^*) and *w^1118^* mutant flies were treated with vehicle or 20 mM α-methyl-tryptophan (αMeW) for 40 h. In vehicle-fed flies, 5-HT was significantly higher in the *w^1118^* mutants compared to wildtype flies (two-way ANOVA, Adj. *R*^2^ = 0.891, *F*_3,20_ = 63.549, *p* < 0.0001; Tukey post hoc for genotype, *p* < 0.01; *n* = 6). Additionally, αMeW significantly lowered the amount of 5-HT in the heads of both wildtype and *w^1118^* mutants (Tukey post hoc for treatment, *p* < 0.0001); (**b**) wildtype Canton-S (*w^+^*) and *w^1118^* mutant flies were fed vehicle or 20 mM αMeW and trained with a single trial. Acquisition in vehicle-fed wildtype and *w^1118^* mutants were significantly different, however there was no effect of αMeW treatment on one-trial learning (two-way ANOVA, Adj. *R*^2^ = 0.519, *F*_3,36_ = 15.05, *p* < 0.0001; Tukey post hoc, *p* < 0.05; *n* = 10). In panels a and b, groups that do not share letters above the box plots are significantly different from each other according to the Tukey post hoc test (*p* < 0.05).

Our data suggest that *w^1118^* mutants have complex defects in 5-HT signaling that impair their rate of learning (Figure 6a). One possibility for this defect in 5-HT signaling is that the *w^1118^* mutants are defective in 5-HT synthesis. This hypothesis predicts lower levels of 5-HT in the heads of *w^1118^*, and that reducing levels of 5-HT will mimic the lower rate of learning found in *w^1118^* mutants. We quantified 5-HT levels of wildtype and *w^1118^* mutant flies fed either vehicle or 20 mM α-methyl-tryptophan (αMeW) (Figure 7a). The αMeW compound has been used successfully to inhibit 5-HT synthesis and signaling in *Drosophila melanogaster* [12,64,66]. Vehicle-fed *w^1118^* heads had statistically significant higher levels of 5-HT than vehicle-fed wildtype heads (Figure 7a; two-way ANOVA, Adj. *R*^2^ = 0.891, *F*_3,20_ = 63.549, *p* < 0.0001; Tukey post hoc for genotype, *p* < 0.01; *n* = 6), and, as predicted, treatment with αMeW lowered 5-HT levels in both genotypes (Figure 7a; Tukey post hoc for treatment, *p* < 0.0001). Surprisingly, wildtype flies treated with 20 mM αMeW, which had reduced 5-HT levels (Figure 7a), did not show reduced performance after one training trial (Figure 7b; two-way ANOVA, Adj. *R*^2^ = 0.519, *F*_3,36_ = 15.05, *p* < 0.0001; Tukey post hoc, *p* < 0.05; *n* = 10). These data suggest that the learning phenotype found after reducing *white* activity is not due to a generalized reduction in 5-HT levels. Instead, the altered performance of *w^1118^* flies given different doses of 5-HTP and L-dopa is likely due to a lack of biogenic amine signaling efficacy. 

**Figure 8:**
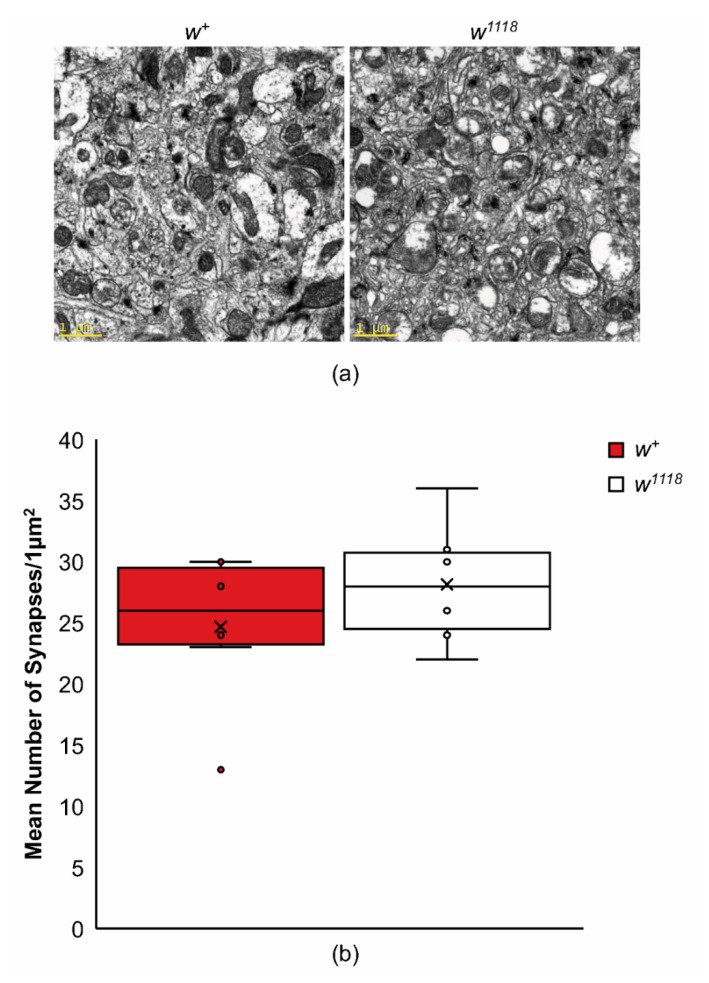
The average number of synapses are not significantly different between wildtype and *w^1118^* mutants. (**a**) Representative micrographs of wildtype Canton-S (*w^+^*) and *w^1118^* mutants in an area adjacent to the mushroom body calyces are shown. Scale bar = 1 mm. (**b**) The average number of central synapses were quantified in wildtype and *w^1118^* mutants; *w^1118^* mutants were not significantly different from wildtype (*t*_10_ = 1.04, *p* = 0.32, *n* = 6).

One possible explanation for the reduced rate of learning and efficacy of 5-HT and dopamine signaling in *w^1118^* mutants is a reduction in synapse numbers. Cholesterol has been shown to modify synapse numbers in *patched* mutants and wildtype flies fed a high-cholesterol diet have more synaptic connections per unit area [67]. We examined the number of synapses in *w^1118^* and wildtype controls in an area that included the mushroom body calyxes (Figure 8). In these experiments, there were no significant differences in the number of synaptic connections found between *w^1118^* and wildtype flies (Figure 8; *t*_10_ = 1.04, *p* = 0.32). These data suggest that the reduction in the efficacy of biogenic amine signaling is not due to a reduced number of synaptic connections in *w^1118^* mutants.

## 3. Discussion

The *white* gene of *Drosophila* has a prodigious history, leading to several fundamental discoveries in genetics [68,69]. Recently, mutants of *white* have been found to have several phenotypes involved in complex behaviors [9,10,11,12,13,14,16,70]. Several of these behaviors are believed to be due to changes in 5-HT signaling in these mutants [12,14]. In this study, we have demonstrated that *white* mutants have a slower rate of learning for olfactory associative short-term memories (Figure 1a). The *white* gene is both necessary and sufficient for the wildtype acquisition of memory (Figure 1b–d). Known binding partners of the white half transporter, scarlet and brown, have normal acquisition of these associative memories, suggesting that for its role in learning, white might be homodimeric, or it partners with other *Drosophila* ABCG transporters such as CG17646 and CG3164 (Figure 2). Several ABCG transporters, including CG17646, are involved in lipid transport and perhaps in cholesterol homeostasis [18,52,71]. 

This study suggests that dietary cholesterol plays a role in olfactory learning for the *w^+^* wildtype, which is possibly mimicked by *w^1118^* mutant flies (Figure 4a). High-cholesterol diets improve learning compared to low-cholesterol diets for wildtype flies, similar to low dietary cholesterol in the *w^1118^* mutants. Higher dietary cholesterol might impair learning in these mutants, though our results were not statistically significant. These differential responses to cholesterol support a role for white in cholesterol homeostasis. Consistent with this role, *w^1118^* flies have altered levels of cholesterol and cholesterol esters (Figure 4c–e), and have increased freeze tolerance (Figure 5)—a phenotype associated with increased cellular cholesterol levels [59]. However, we do not believe this role in homeostasis necessitates a direct cholesterol transport function for white. 

The precise roles of 5-HT and dopamine signaling in this *w^1118^* learning phenotype are less clear. While small amounts of the 5-HT precursor, 5-HTP, can rescue learning in *w^1118^* mutants, increasing 5-HT has an overall quiescing effect on wildtype learning (Figure 6a). In addition, feedings of L-dopa alone and combined with 5-HTP partially recover normal learning performance (Figure 6b,c). The white mutants might, therefore, alter the lipid composition and/or distribution of lipids in a manner that reduces 5-HT and dopamine signaling efficiency involved with the learning phenotype.

### 3.1. Known Biochemical Activities of white and Cellular Locations 

White is best known for its role in the fly eye. The white gene encodes the half of an ABCG transporter for the precursors of eye pigments [22,72]. However, the ability of white to form multiple functional heterodimers indicates that this protein could potentially affect many different cellular processes. White has been implicated in the transport of pyruvate, riboflavin, xanthine, guanosine, and zinc [46,47,73,74,75,76]. In most of these cases, white is involved in concentrating these compounds into vesicles [46,47,74,75]. White has also been proposed to facilitate the transport of precursors for biogenic amines in *D. melanogaster*, including tryptophan [74]. This amino acid is the precursor for ommochrome pigments in the eye and 5-HT, a monoamine neuromodulator involved in learning and memory. However, serotonin is synthesized cytoplasmically [77] and, as an efflux transporter, it seems unlikely that white is responsible for directly bringing tryptophan into the cell. There is some evidence of its ability to transport tryptophan in the Malpighian tubules of *Drosophila*, although this transport was independent of the cytoplasmic pool of tryptophan and several other studies have indicated that kynurenine and 3-hydroxykynurenine are the tryptophan metabolites transported into pigment granules by white [22,74,78]. 

The *white* gene is expressed at various levels in many tissues, including the brain [36,79]. This ABCG transporter is expressed in both adults and larvae. While mRNA levels are low in the brain itself, the whole head has five times higher levels than the brain, suggesting that some neural effects of white may originate in head tissues surrounding the brain. Most expression is in adult/larval tubules and the larval fat body [47,79]. The intercellular localization of a white: DsRed fusion proteins expressed in S2 and in COS cells appears to be endosomal, consistent with a vesicular localization in pigment cells and in the Malpighian tubules [47,70,72].

### 3.2. The Role of white in Behavior 

Mutants of *white* have numerous behavioral phenotypes, including some that are due to poor visual acuity [6,7,9,11,12]. There are also several behavioral phenotypes that are non-visual [13,14,15,16]. Some of these non-visual behaviors are influenced by defects in 5-HT signaling. For example, operant place memory has been tested in an aversive heat box assay. The *white* mutant has an attenuated memory in this assay that is replicated with the 5-HT synthesis inhibitor, α-methyltryptophan, but not by the dopamine synthesis inhibitor, α-methyltyrosine [12]. Other 5-HT-dependent behavioral changes seen with mutation and misexpression of *white* mutants include an increase in the sexual arousal of male flies, leading to male–male courtship [14,15], and an increase in the variability of phototactic behaviors between individual flies [66]. Wildtype phototactic behavior is restored to *w^1118^* mutants by consumption of the precursor of 5-HT, 5-hydroxytryptophan (5-HTP) [66]. As with operant learning in the heat box assay, the mutant behavior is mimicked by α-methyltryptophan feeding.

### 3.3. A Role for white in Olfactory Learning 

Previous work with *white* in short-term olfactory memory looked at performance after asymptotic aversive training and did not find a difference from wildtype. We utilized the short program to deliver discrete training trials to *w^1118^* mutants, which allowed us to examine the acquisition rate for this associative memory [41,43]. Acquisition curves are especially helpful for differentiating between learning defects and memory defects, because flies with different rates of learning may still have similar performance indices for short-term memory in the long program that uses optimal learning conditions. Since *white* is defective in the short program of a single shock with 10 s of odor but not the long program, *white* appears to be required for learning, rather than short-term memory (Figure 1a). Sensory defects were tested through naïve odor avoidance and shock avoidance, with no significant difference found between *w^+^* and *w^1118^* flies on regular diets (Supplementary Table S1). However, *w^1118^* mutants showed an increased shock avoidance to lower voltages than normally used in olfactory training [11]. This increased sensitivity to the unconditioned stimulus (US) may predict an increase in the magnitude of performance in the olfactory T-maze [41], which we did not see.

### 3.4. Dominance and Gene Dose Sensitivity

The null *w^1118^* and hypomorphic alleles, *w^a^* and *w^co^*, have approximately 50% of the short-term olfactory memory of wildtype Canton-S flies and are statistically no different in terms of short-term memory with a single shock, despite a gradient of expression within the eyes (Figure 1b). Moreover, *w^1118^* is dominant for this learning phenotype in heterozygous females (Figure 3), but increasing *white* expression by adding two mini-*white* transgenes or a large duplication of *white* to the *w^1118^* mutant flies rescues the wildtype learning phenotype (Figure 1c,d). These data strongly support the hypothesis that the learning deficit in *w^1118^* flies is due to the loss of *white* and that *white* is haploinsufficient for learning. A very high level of *white* expression was also required to rescue the *w^1118^* recovery from anoxia phenotype [80]. Furthermore, the rescue of the loss-of-function of *white* in male copulation success is sensitive to dosage and requires high expression levels of *white* [81]. Together, it appears that high levels of *white* expression are needed to support several behaviors, consistent with general haploinsufficiency for these behaviors.

### 3.5. ABCGs with Cholesterol and Other Lipid Functions

We sought insight into the possible biochemical functions of white from the activities of homologs in other species. Specifically, the human ABCG1, ABCG4, ABCG5, and ABCG8 are all involved in regulating cholesterol levels. All are sterol efflux proteins, and while some function as likely homodimers, others function as heterodimers. ABCG1 is a sterol-induced gene with ubiquitous expression and multiple splice variants, resulting in two major protein variants [27]. The protein has been localized to endosomes [28] and involved with intracellular sterol transport. Both homodimers and heterodimers have been found possible for ABCG1. Mutation of ABCG1 protects mice against obesity in a high-cholesterol diet (1%). Mice with an ABCG1 null mutation have a normal life span, as do *w^1118^ Drosophila* null mutants under standard rearing conditions [31,82]. ABCG1 null mice were also found to have significantly higher locomotor activity and energy expenditure, along with lower fasting plasma lipid levels [82]. Hence, ABCG1 appears to be a regulator of cholesterol metabolism that may couple diet and activity in mammals.

ABCG4 is primarily expressed in the CNS, and homozygous mouse knockout mutants have defects in contextual associative fear memory (tone and foot shock pairings) but not in the Morris Water Maze [30]. ABCG4 can be a homodimer or heterodimer, and it has been found to be co-expressed with ABCG1 in both neurons and astrocytes, where it regulates cholesterol homeostasis [30,83]. Mouse knockouts of both ABCG1 and ABCG4 increase oxysterols in the retina and the brain. While both ABCG1 and ABCG4 have high homology with *white*, it is important to note that neither ABCG1 nor ABCG4 can replace the *Drosophila white* gene for recovery of eye pigmentation in *white* null flies, even though ABCG1 is also highly expressed in the eyes of mammals [29].

Human mutations in ABCG5 and ABCG8 contribute to the autosomal recessive β-sitosterolemia, a metabolic lipid disorder that causes hypercholesterolemia due to overabsorption of dietary sterols [32,84]. This disorder is found in many genetic backgrounds and from many different amino acid substitutions within each population [85], but always from a homozygous mutation of a single gene, ABCG5 or ABCG8. These ABCGs are needed for efflux of dietary sterols from intestinal epithelia to the lumen of the gut and from liver cells to the bile duct. The highest expression levels of ABCG5 and ABCG8 are in the intestines and liver, with transcriptional expression upregulated by cholesterol feeding [32].

White, like all ABCGs, is a half-transporter that might homodimerize or heterodimerize with other *Drosophila* ABCGs. If brown or scarlet, the known binding partners of white, are involved in the learning phenotype, we would expect the *bw^1^* or *st^1^* mutants to have poor learning for single shock learning, like the *w^1118^* mutant. In contrast, we found that these mutants had wildtype levels of learning (Figure 2a), necessitating the search for new potential binding partners. Vertebrate homologues of *w^1118^* can be homodimers or heterodimers with other ABCG half transporters, so the possibility exists that the acquisition defect depends on more than one pairing. *Drosophila melanogaster* has 15 ABCG transporters. We investigated five of these ABCGs for learning defects (*CG3164*, *CG4822*, *CG17646*, *CG9663*, and *Atet*), based upon homology and relatedness to ABCG1/4, as well as adult head expression [50,86]. These are only some of the potential partners that might have exhibited poor learning and there is still the possibility of homodimerization of white similar to ABCG1 or ABCG4. Of particular interest were the mutants with demonstrated roles in cholesterol and lipid regulation. An insertional mutation in *CG17646*, an ABCG1-like protein, resulted in an increased triglyceride content in the fly [82]. An *ATP transporter expressed in the trachea* (*Atet*) is involved in neurogenesis and vesicle-mediated ecdysone release [34] in the prothoracic gland. *CG9663* is transcriptionally regulated by *Drosophila* hormone receptor 96 (DHR96), a nuclear receptor that responds to cholesterol levels [35]. 

The most promising potential binding partners for *white*, according to significance in the learning phenotype, are *CG3164^MI06431^* and *CG17646^MI04004^* (Tukey (HSD) post hoc, *p* < 0.05). *CG3164* is highly expressed as a maternal transcript [87] and has been found in a screen for actively translated RNAs during embryogenesis [88]. Our female heterozygotes of *w^+^*/*w^1118^* were produced from wildtype females but exhibited the mutant phenotype, suggesting that *white*, and possibly its binding partner for this learning phenotype, is not maternal (Figure 3a,b). The *CG17646* hypomorph has no maternal expression, high RNA expression in the central brain of embryos [50], and high RNA expression in the adult fat body [86]. In addition, *CG17646* mutants have been found to have 1.5–1.7× higher than normal levels of triglycerides in a whole body assay [82], suggesting that they function to regulate lipid metabolism. Of note is the lower MCH and 3-OCT avoidance for all new ABCG mutants that may weaken the associative strength of the shock-paired odor (Supplementary Table S1). Yet, a similar reduction in odor avoidance did not impact learning in *Atet* mutants and normal odor avoidance was present for *w^1118^* mutants. It remains possible that the new ABCG mutants with poor acquisition have defects that alter learning via a processing or sensory deficit, or that these learning defects are not due to the loss of ABCG function.

### 3.6. Cholesterol Regulation in Drosophila

Sterol metabolism can be investigated with relatively good experimental control in *Drosophila* since they are cholesterol auxotrophs [53]. Although a sterol-free diet is lethal, stalling development in larval stages [89], a simple minimal food of bactoagar, yeast, and sucrose has enough yeast-derived sterols to support life and yields flies of normal mass (Figure 4b) with no obvious anatomical differences. We showed that *w^+^* and *w^1118^ Drosophila* grown on this low-cholesterol food and the same food with 0.1 mg/mL cholesterol have differently regulated learning and cholesterol homeostasis that is dependent on dietary cholesterol (Figure 4a–d). Higher levels of cholesterol were necessary in *w^+^* flies for better learning, while low cholesterol was better for *w^1118^* mutant flies (Figure 4a). 

Cholesterol homeostasis in flies depends upon the nuclear receptor DHR96 [35,90]. DHR96 directly binds cholesterol and controls the activity of many genes regulated by low and high dietary cholesterol, including the *dABCA1*, *CG32186*, and *CG9663* ABC transporter genes [35,90]. *DHR96* mutants had higher levels of cholesterol, and high-cholesterol diets in flies mimicked the transcriptional profile of the genes up/downregulated in the *DHR96^1^* mutant raised on standard food [35,90]. This result suggests that DHR96 is necessary for keeping cholesterol levels down by controlling genes needed for changing dietary conditions. The Nieman Pick 1b transporter is responsible for dietary cholesterol uptake and is dysregulated in the *DHR96^1^* mutant, which contributes to the lethality of the *DHR96* mutants on low-cholesterol diets [35]. The transcriptional profile of *white* was not reported to have changed in response to cholesterol or in the *DHR96^1^* mutants, even though *w^1118^* mutants have defects in cholesterol levels, tolerance to freezing temperatures, and behavioral responses to dietary cholesterol [35,90].

### 3.7. Differences in the Cholesterol and Cholesterol Ester Levels of w^1118^

Given the roles of many ABCGs in the regulation of cholesterol, we used LC-MS to measure cholesterol and cholesterol ester levels for wildtype and *w^1118^* mutant flies fed controlled diets of either low or high cholesterol. Our data showed a statistically significant increase in cholesterol levels in the mutant flies compared to the wildtype flies when given a low-cholesterol diet (Figure 4c), as well as a higher, yet insignificant, level of cholesterol in the mutant flies when fed a high-cholesterol diet (Figure 4d). This suggests a change in cholesterol homeostasis and, possibly, lipid membrane composition, rather than general metabolism. A difference in cell membrane composition is supported by the mortality of *w^1118^* mutants subjected to subfreezing temperatures being lower than that of the wildtype, and this being rescued to wildtype percentages by the mini-*white* transgene NP1131 (Figure 5). This increased freeze tolerance in the *w^1118^* flies suggests an increase in membrane fluidity that would occur from higher levels of cholesterol [59,91]. 

We looked at olfactory learning in *w^+^* and *w^1118^* flies fed low- and high-cholesterol diets and found similarities between the dietary effects on learning and cholesterol esters, suggesting that cholesterol homeostasis may play a role in learning. While a diet of high cholesterol improved learning for the wildtype flies, mirroring the higher levels of cholesterol and 18:1 cholesterol ester measured in similarly fed flies, the *w^1118^* flies had a higher learning performance index when fed a low-cholesterol diet, which mimicked the higher levels of all measured cholesterol esters fed similarly (Figure 4a,e). Notably, all performance indices were low compared to usual wildtype learning, suggesting that these diets represented suboptimal diets. In addition, differences in learning do not appear to be a result of changes in synapse number between genotypes (Figure 8). This suggests a role for cholesterol and/or cholesterol esters in another aspect of signaling, such as lipid raft stability or synaptic dynamics.

### 3.8. Roles of Cholesterol in Synaptic Signaling

We found that a reduction in dietary cholesterol can reduce the performance of wildtype flies in one-trial olfactory learning. Cholesterol is required for synaptogenesis, and reduced levels of cholesterol may lead to fewer synapses [67,92]. Since *w^1118^* mutants had higher levels of cholesterol, one possibility would be that these mutants had too many synapses for efficient learning. However, *w^1118^* mutants do not have significantly altered numbers of synapses, suggesting that changes in synaptogenesis are not responsible for the slower rate of learning. 

Cholesterol is found at high levels within synapses and is important for maintaining synaptic signaling, with both pre- and postsynaptic functions [93,94,95]. Most work on the role of cholesterol in neuronal functions has relied upon the pharmacological depletion of cholesterol from plasma membranes. This depletion leads to reductions in evoked vesicle release [95,96,97]. It has been proposed that the decrease in the evoked release is due to a need for cholesterol in action potential propagation [95]. Synaptic vesicles are also rich in cholesterol, and the depletion of cholesterol levels from vesicle pools impairs synaptic vesicle recycling [98,99]. Cholesterol depletion can also affect the strength of synaptic signaling by modulating neurotransmitter reuptake transport proteins [100]. For example, the mammalian DAT and SERT monoamine transporters are normally localized in cholesterol-rich rafts, both internally and on the cell surface, and the depletion of cholesterol results in a reduction in the number of these transporters on the cell surface, leading to a reduced termination in neurotransmitter signaling [101,102,103]. The activity of these transport proteins is also likely modified by direct cholesterol binding [104]. Together, these studies demonstrate a need for cholesterol in specific membrane pools for the effectiveness of different presynaptic functions. 

Cholesterol can also alter GPCR signaling in the lipid bilayer through direct interaction with GCPRs and/or by altering the lipid ratio in the membrane [105,106]. GPCRs are embedded in cholesterol-rich lipid rafts, which allow the receptors to interact with G-proteins and other associated proteins and maintain functional conformations [107]. Alterations in the lipid composition can disrupt signaling from receptors despite adequate ligands. A good example of this is the mammalian 5-HT1A receptor. 5-HT1A is a G_α(i/o)_-coupled GPCR that, in primary human neuronal cultures, reduces cAMP and PKA signaling following 5-HT binding, leading to attenuation of S^133^-CREB phosphorylation, as well as reduced T^185^/Y^187^-ERK2 phosphorylation in the MAPK pathway [54]. Sequestration of cholesterol with methyl-β-cyclodextrin in human primary neuron cultures was able to counteract the inhibition of ERK2 and CREB phosphorylation by 8-OH-DPAT agonism of 5-HT1A, demonstrating a positive role for cholesterol in 5-HT1A signaling [54]. Cholesterol depletion can reduce GPCR signaling through the loss of direct binding, reduced organization of signaling rafts, or impaired trafficking [105,106,107]. The effect of elevated cholesterol on GPCR efficacy is much less clear and may be subtler. However, it is likely that a reduction in GPCR signaling efficacy or presynaptic functions could also occur if the membrane distribution of cholesterol is altered, even when total cholesterol levels are higher. 

### 3.9. white and Biogenic Amine Signaling in Olfactory Learning

Wildtype levels of learning were partially rescued in our *w^1118^* mutant flies by increasing either 5-HT or dopamine levels (Figure 7). Olfactory learning in *Drosophila* requires both serotonin and dopamine [62,63]. Dopamine has multiple complex roles in olfactory learning, including encoding the electric shock unconditioned stimulus, predicting the US, reinforcing memory, and active forgetting [62,108,109,110]. Dopamine has also been reported to function in appetitive memory [111,112]. Substantial serotonergic innervation and expression of 5-HT receptors exists in the olfactory learning circuitry of *Drosophila*, notably, the serotonergic dorsal-paired medial neurons (DPMs) that innervate the MBs both pre- and post-synaptically [113]. 5-HT signaling is involved in short-, intermediate-, and long-term memory [63,114]. While the relationship between 5-HT signaling and acquisition is not completely clear, all 5-HT receptors are needed for short-term olfactory memory [63]. The 5-HT1a and 5-HT1b receptors are heavily expressed in MBs [115,116]. 

Due to the fact that other *white* mutant behavioral phenotypes have been mimicked by reduction of 5-HT [12,14,66] and 5mM 5-HTP was able to rescue the learning defect in *w^1118^* flies (Figure 6a), global pharmacological inhibition of 5-HT synthesis was investigated for effects on learning. We found that feeding flies αMeW to reduce 5-HT levels in *w^+^* flies reduced the levels of 5-HT in their head but did not mimic the *w^1118^* one-trial learning phenotype (Figure 7b). Moreover, *w^1118^* flies fed vehicle showed a significant elevation in 5-HT levels compared to vehicle-fed *w^+^* flies (Figure 7a), suggesting a reduction in 5-HT signaling efficacy rather than synthesis might contribute to the learning phenotype. Though white has been hypothesized to alter 5-HT levels via the transport of precursors for 5-HT, this hypothesis predicts that mutants in either the *brown* or *scarlet* co-transporters would also have a defect in learning [12,36]. Yet, the learning phenotype in this study was independent of *brown* and *scarlet* (Figure 2a). The absence of phenotypes for *brown* and *scarlet* mutants suggests that white is paired with itself or other ABCG half-transporters for its function in learning, and this function is independent of 5-HT synthesis. If 5-HT signaling is not altered at the level of 5-HT synthesis, it might be altered by relatively subtle changes to presynaptic activity, receptor availability or stability, or an additive contribution from some combination of pre- and post-synaptic signaling deficiencies. Altered cholesterol homeostasis may offer a possible explanation for these results. 

### 3.10. Possible Mechanisms for L-Dopa and 5-HTP Rescue of Wildtype Learning Phenotype

Although there are several possibilities for how *white* may be modulating the rate of olfactory learning through a role in cholesterol homeostasis, perhaps the most direct scenario is that the white protein dimerizes with one or more other ABCGs to transport cholesterol, or a metabolite of cholesterol, into the proper vesicle, such as an endosome, for storage and/or delivery. Disruption of this transport would alter the composition of lipid membranes and the lipid rafts within them that are crucial for efficient biogenic amine signaling. Moreover, this hypothesis predicts that other mutations and treatments that alter the membrane composition of *Drosophila* neurons could phenocopy or rescue the *w^1118^* learning defect, similar to the effect of reducing dietary cholesterol. *Drosophila* that are deprived of cholesterol are known to upregulate sphingolipids as replacements and change the lipid raft integrity needed for signaling, though the central nervous system is relatively good at maintaining sterols compared to other tissues like the fat body [117]. Understanding the precise biochemical function of white that drives changes in cholesterol levels and sensitivity should provide important insights into the role of in vivo membrane lipid dynamics in regulating neural signaling during learning. 

## 4. Materials and Methods

### 4.1. Drosophila Stocks and Genetics

Fly stocks and crosses were maintained in a 25 °C incubator with a 12:12 h light:dark cycle and cultured on cornmeal medium, except for experiments involving dietary cholesterol. Cholesterol feeding experiments used a standard media of 50 g sucrose (S0389, Sigma-Aldrich, St. Louis, MO, USA), 17 g yeast (Active Dry Yeast, Fleischmann’s Yeast), and 15 g *Drosophila* Agar—Type II (66–105, Genesee Scientific, San Diego, CA, USA ), with or without 0.1 mg/mL cholesterol (C3045, Sigma-Aldrich, St. Louis, MO, USA) as previously published [118]. Powdered cholesterol was dissolved in 100% ethanol and added (1 mL per 40 mL food) to media during cooking and after cooling to 60 °C. Ethanol was added to control food to account for possible effects on growth, development, or behavior from residual ethanol in the diet. Fifteen females and 10 males of either wildtype Canton-S or *w^1118^* genotypes were placed into bottles with 40 mL of media and brooded every 4–5 days for a total of three sets of bottles.

Most stocks were obtained from the Bloomington *Drosophila* Stock Center (BDSC, Bloomington, Indiana, USA) and are listed in Table 1. MiMIC lines from BDSC were previously constructed [119]. The c739 and NP1131 GAL4 lines were a gift of Ron Davis (Scripps, Jupiter, FL, USA) and were brought together by meiotic recombination.

All transgenic fly strains were backcrossed to the Roman Lab’s Canton-S background. This backcross procedure began by using balancer chromosome lines to exchange the chromosomes not bearing the transgene with chromosomes from the Roman Lab Canton-S background. The transgenes were then crossed for a minimum of six generations into either a *w^1118^*[CS10] or a *y^1^*[CS10] stock, both of which had been previously outcrossed in the Roman Lab Canton-S lineage for 10 generations. After the outcrossing was complete, the X chromosome was replaced using a *w^+^* balancer line to ensure a fully wildtype Canton-S X- chromosome. For behavioral experiments, 1–4-day-old *Drosophila* were collected under mild CO_2_ anesthetization at least 1 day prior to each experiment. Wildtype white (*w^+^*) or mutant (*w*^−^) alleles on the X chromosome are specified for each experiment. Associative learning assays used both males and females, except for experiments that specifically tested *w^+^*/*w^1118^* heterozygotes. In that case, only females were used. No difference was seen in the performance scores of females alone versus males and females combined with the same homozygous white allele background.

### 4.2. Pharmacology

*Drosophila* were collected at 0–3 days post-eclosion and treated by feeding them 2% sucrose, with or without drug treatment, at the concentrations specified in the text [65]. After CO_2_ anesthetization, flies were placed into vials with half a Kimwipe saturated with 1 mL of solution for 40–48 h, depending on the experiment. Hydroxy-L-tryptophan (H9772 Sigma), 3,4-Dihydroxy-L-phenylalanine (L-DOPA, D9628 Sigma), and αMethyl-DL-tryptophan (M8377 Sigma) were all delivered using this method. Flies were returned to regular food 30–60 min prior to behavioral experiments for the flies to clean themselves of sucrose that may interfere with behavior.

### 4.3. Olfactory Classical Conditioning Assay

A 3-Octanol (218405 Sigma-Aldrich, St. Louis, MO, USA) and 4-Methylcyclohexanol mixture of cis and trans (153095, Sigma-Aldrich, St. Louis, MO, USA) were diluted in light mineral oil (330779, Sigma-Aldrich, St. Louis, MO, USA) for olfactory conditioning. A quantity of 20 μL 3-Octanol (3-OCT) was diluted in 20 mL of light mineral oil and odor balanced with 12–22 μL of MCH at regular intervals to account for variation in experimental conditions day-to-day.

Flies were collected at 0–4 days post-eclosion and either transferred to fresh food for the assay or to vials containing a half Kimwipe (approximately11.4 cm × 10.7) saturated with 1 mL of 2% sucrose, with or without drug treatment. Flies were transferred to fresh food 30–60 min prior to each assay. All flies were placed in the testing room at 25 °C and 60–75% humidity with dim red light 30–60 min prior to being assayed.

Classical olfactory conditioning was performed using a T-maze similar to previous studies [41,43,120]. For single-trial learning, 1–5-day-old flies were trained using 10 s single exposures to MCH and 3-OCT, alternatively paired with a 1.25 s 90 V shock and 30 s of fresh air intervals between odors. Flies were then tapped into an “elevator”, allowed to rest for 1 min, and exposed to a choice of each odor for 2 min to test for associative short-term memory (STM). For the acquisition curve, a variation of the short program was used. For all groups in this variation, odor was delivered for 60 s and 1.25 s 90 V shocks delivered towards the end of the exposure, spaced 5 s apart. Flies were then rested for 1 min and tested for 2 min, as with the 10 s single exposure assays. The half performance index (PI) was calculated for each odor as follows: ((# of flies in unpaired odor tube, CS-)—(number of flies in paired odor tube, CS+))/(total number of flies in both tubes). The half PIs were then averaged to give a full PI and account for learning differences between odors [41].

Odor and shock avoidance were tested for new phenotypes. For naïve odor avoidance, odorants were presented in the same conditions and using the same concentrations used for the conditioning assay opposite pure mineral oil in a T-maze. The only exception was for odor avoidance with and without 2 mg/mL L-dopa, tested with similar concentrations but later at the University of Mississippi. Flies were exposed for 2 min and collected as before. Half of the exposures used odorant on the right and the other half on the left to account for any directional bias from other unaccounted for stimuli in the testing room. For shock avoidance, one arm of the T-maze was replaced with a shock tube and both arms connected to pure mineral oil for consistent odor on each side. For 2 min, flies were exposed to the T-maze while 90 V was delivered every 5 s.

### 4.4. Cholesterol Treatments

Fifteen female and 10 male wildtype Canton-S or *w^1118^* flies were allowed to breed for 4–5 days and oviposit embryos on either 40 mL of control media (5% sucrose, 1.7% yeast, and 1.5% Bacto^TM^ agar) or control media with 0.1 mg/mL cholesterol, as described. Control media provides negligible amounts of cholesterol solely through the yeast; however, this is sufficient for growth and development of *Drosophila* [35,121]. Four groups were cultivated: Canton-S with low cholesterol, Canton-S with high cholesterol, *w^1118^* with low cholesterol, and *w^1118^* with high cholesterol. There were 4–5 bottles for each group. Parent flies were transferred twice, for a total of 3 sets. For olfactory learning, 0–4-day-old flies were trained and tested as usual with the short program. For each set, 3 subsets of 20 females and 20 males were collected from each group and weighed. The 9 subsets for each sex of each group were averaged and analyzed for differences in mass between treatment groups. For cold tolerance, 2–5-day old male and female flies were harvested, placed in snap cap tubes, allowed to recover from CO_2_, and used immediately for cold tolerance experiments.

### 4.5. Cholesterol and Cholesterol Ester Quantification

Canton-S and *w^1118^* flies (30 males and 30 females) were fed either a low- or high-cholesterol diet and whole flies were frozen and stored at -80 °C. LC-MS analysis services for cholesterol and cholesteryl esters were provided through Wayne State University Lipidomics Core Facility. Flies were shipped on dry ice to the Lipidomics Core Facility for LC-MS analysis of cholesterol and cholesteryl esters levels using a standard operating procedure based on published methods [122,123] and reported as ng per mg of total protein.

### 4.6. Freeze Tolerance

Freeze tolerance assays were adapted from the work of Shreve et al. [59]. Twenty flies of each group were placed in a snap cap, 15 mL test tubes, and allowed to recover. Flies were submerged in a chilled bath at −5 °C and kept submerged for 120 min. Flies were then removed and transferred to a fresh *Drosophila* vial with standard media and placed into a 25 °C incubator. After twenty-four hours, flies were scored for survival. Flies were considered to have survived if they were able to right themselves after a gentle tap. Mortality percentage was calculated as follows: ((total number of flies—total number of flies alive)/total number of flies) ∗ 100.

### 4.7. Quantifying Synapse Numbers

Wildtype Canton-S and *w^1118^* flies were prepared for electron microscopy as previously outlined [67]. The collected transmission electron micrographs were analyzed with Fiji [124]. The metadata relating to the scale used during the imaging process were preserved in the images. The “Grid” tool in Fiji was used to generate a grid with area = 1 µm^2^ over each image. Using this grid, tiles with area = 1 µm^2^ were selected for analysis. For each genotype, 6 tiles were used resulting in a total of 12 tiles. Within each tile, the “Cell Counter” plugin was used to manually count each synapse that was visible. The data that were collected were then transferred to Microsoft Excel for statistical analysis.

### 4.8. HPLC for 5-HT

Six vials each of 40 Canton-S and *w^1118^* flies (20 males and 20 females) were treated with either 20 mM αMethyl-DL-tryptophan for 40 h or 50 mM 4-Chloro-L-phenylalanine for 45 h in 2% sucrose alongside controls in 2% sucrose alone. Flies were frozen in liquid nitrogen and heads were separated with sieves on liquid nitrogen before storage at −80 °C. Flies were shipped on dry ice to the Vanderbilt Neurochemistry Core for HPLC analysis of 5-HT levels as ng per ml of total protein.

### 4.9. Data Analysis

Normality of the data was tested with the Shapiro–Wilk test [125]. For parametric data, analysis of variance (ANOVA) was used to determine model effect sizes and overall significance in olfactory conditioning assays. Post hoc analysis was done with Tukey for multiple comparisons and Dunnett’s for experimental comparisons to control. Student’s t-tests were used when only two groups were appropriate for comparison because effects were expected to be independent from other groups. *p* values < 0.05 were considered statistically significant. For non-parametric data, significance between groups was calculated using the Kruskal–Wallis test and reported as a *K* statistic with Dunn’s post hoc analysis [126]. These statistics were calculated using XLSTAT version 19.5 and 2020.5.1 (Addinsoft, New York, NY, USA). For the box and whisker plots, the middle line in each box represents the median, the X is the mean, the upper box is range of the 3rd quartile, while the lower box represents the range of the 2nd quartile. In these box and whisker plots, the error bars represent the range excluding outliers. In the bar graphs, error bars for all analyses are standard error of means (*SEM*).

## Figures and Tables

**Table 1:** Sources of *Drosophila* stocks.

Fly Stock Used	Annotation in Text	Identifiers
w[1118];Dp(1;3)DC050,PBac[y[+mDint2] w[+mC] = DC050]VK00033	*w^1118^*;+,wDup	BDSC 30234
*bw^1^*; *st^1^*	*bw^1^; st^1^*	RRID:BDSC_686
*w^a^*	*w^a^*	RRID:BDSC_48
*w^co^*, *sn^2^*	*w^co^*, *sn^2^*	RRID:BDSC_153
w[1118]; P[w[+mW.hs] = GawB]Hr39[c739], P[GawB]NP1131	*w^1118^*;c739, NP1131	RRID:BDSC_7362, RRID:FBti0034208
MiMIC lines and y[1] w[*]; Mi(y[+mDint2] = MIC)CG3164[MI06431]	*CG3164^MI 06431^*	RRID:BDSC_42392
y[1] w[*]; Mi(y[+mDint2] = MIC)ND-15[MI10825] CG3164[MI10825]/SM6a	*CG3164^MI 10825^*	RRID:BDSC_55545
y[1] w[*]; Mi(y[+mDint2] = MIC)ND-15[MI13074] CG4822[MI13074]	*CG4822^MI 13074^*	RRID:BDSC_58020
y[1] w[*]; Mi(y[+mDint2] = MIC)CG17646[MI04004]	*CG17646^MI 04004^*	RRID:BDSC_42302
y[1] w[*]; Mi(y[+mDint2] = MIC)CG9663[MI11447]	*CG9663^MI 11447^*	RRID:BDSC_56321
y[1] w[*]; Mi(y[+mDint2] = MIC)Atet[MI01881]	*Atet^MI 01881^*	RRID:BDSC_37314

## Data Availability

The data presented in this paper are publicly available at: https://egrove.olemiss.edu/pharmacy_facpubs/131 (accessed on 29 November 2021).

## Supplementary Materials

The following are available online at https://www.mdpi.com/article/10.3390/ijms222312967/s1.
