# Supplementary material for: Mutants of the white ABCG Transporter in Drosophila melanogaster Have Deficient Olfactory Learning and Cholesterol Homeostasis
Source: Int J Mol Sci. 2021 Nov 30;22(23):12967. doi: 10.3390/ijms222312967 (PMC8657504; doi:10.3390/ijms222312967)
Supplement: Supplementary file 1 [file ijms-22-12967-s001.zip › ijms-1437452-supplementary.pdf]

International Journal of *Molecular Sciences*

MDPI

Article

Mutants of the *white* ABCG Transporter in *Drosophila melanogaster* Have Deficient Olfactory Learning and Cholesterol Homeostasis

Supplementary Materials

Table S1. Odor and Shock Avoidance Controls.

Experiment	Genotype	Shock Avoidance	OCT Avoidance	MCH Avoidance
40mM 5-HTP Treatment Effects on Naïve Avoidance	**Canton-S (+)**	**0.705 ± 0.035**	**0.462 ± 0.056**	**0.558 ± 0.062**
	Canton-S (+) + 40mM 5-HTP	0.518 ± 0.050*	0.482 ± 0.098	0.514 ± 0.093
	white^{1118}	0.736 ± 0.023	0.247 ± 0.086	0.633 ± 0.072
	white^{1118} + 40 mM 5-HTP	0.659 ± 0.0317	0.260 ± 0.111	0.594 ± 0.066
		n = 10	n = 12	n = 10
L-dopa Treatment Effects on Naïve Avoidance	**Canton-S (+)**	**0.755 ± 0.076**	**0.478 ± 0.050**	**0.255 ± 0.055**
	Canton-S (+) + 2 mg/ml L-dopa	0.743 ± 0.030	0.186 ± 0.058*	0.226 ± 0.062
	white^{1118}	0.867 ± 0.046	0.415 ± 0.029	0.259 ± 0.050
	white^{1118} + 2 mg/ml L-dopa	0.855 ± 0.025	0.430 ± 0.037	0.334 ± 0.027
		n = 4	n = 8	n = 8
Naïve Avoidance of ABCG mutants	**Canton-S (+)**	**0.751 ± 0.024**	**0.544 ± 0.069**	**0.752 ± 0.067**
	Atet $^{MI\ 01881}$	0.735 ± 0.040	0.329 ± 0.052	0.194 ± 0.104*
	CG4822 $^{MI\ 13074}$	0.824 ± 0.014	0.285 ± 0.057*	0.316 ± 0.104*
	CG9663 $^{MI\ 11447}$	0.852 ± 0.023	0.357 ± 0.048	0.326 ± 0.113*
	CG3164$^{MI\ 06431}$	0.835 ± 0.022	0.297 ± 0.068	0.300 ± 0.101*
	CG3164$^{MI\ 10825}$	0.855 ± 0.013	0.287 ± 0.067*	0.347 ± 0.136*
	CG17646 $^{MI\ 04004}$	0.738 ± 0.032	0.291 ± 0.095*	0.401 ± 0.131
	w^{1118}	0.739 ± 0.036	0.611 ± 0.578	0.730 ± 0.065
		n = 10	n = 10	n = 10
Naïve Avoidance of *CG17646* and *w*^{1118} Heterozygotes	**Canton-S (+)**	**N/A**	**0.359 ± 0.070**	**0.802 ± 0.076**
	+; *CG17646* $^{MI\ 04004}$/+	N/A	0.254 ± 0.076	0.803 ± 0.078
	w^{1118}/+; +	N/A	0.399 ± 0.083	0.777 ± 0.079
	w^{1118}/+; *CG17646* $^{MI\ 04004}$/+	N/A	0.351 ± 0.083	0.743 ± 0.096
	w^{1118}; +	N/A	0.428 ± 0.083	0.803 ± 0.062
			n = 8	n = 8
Naïve Avoidance of *CG3164* and *w*^{1118} Heterozygotes	**Canton-S (+)**	**N/A**	**0.570 ± 0.040**	**0.729 ± 0.099**
	+; *CG3164* $^{MI\ 06431}$/+	N/A	0.187 ± 0.121*	0.467 ± 0.172
	w^{1118}/+; +	N/A	0.377 ± 0.133	0.648 ± 0.136
	w^{1118}/+; *CG3164* $^{MI\ 06431}$/+	N/A	0.303 ± 0.107	0.475 ± 0.167
	w^{1118}; +	N/A	0.589 ± 0.039	0.692 ± 0.112
			n = 10	n = 10

*Denotes a significance difference from wildtype Canton-S control in bold (Two-sided Dunnett Analysis of difference between Canton-S wildtype control and experimental genotype with 95% confidence interval).

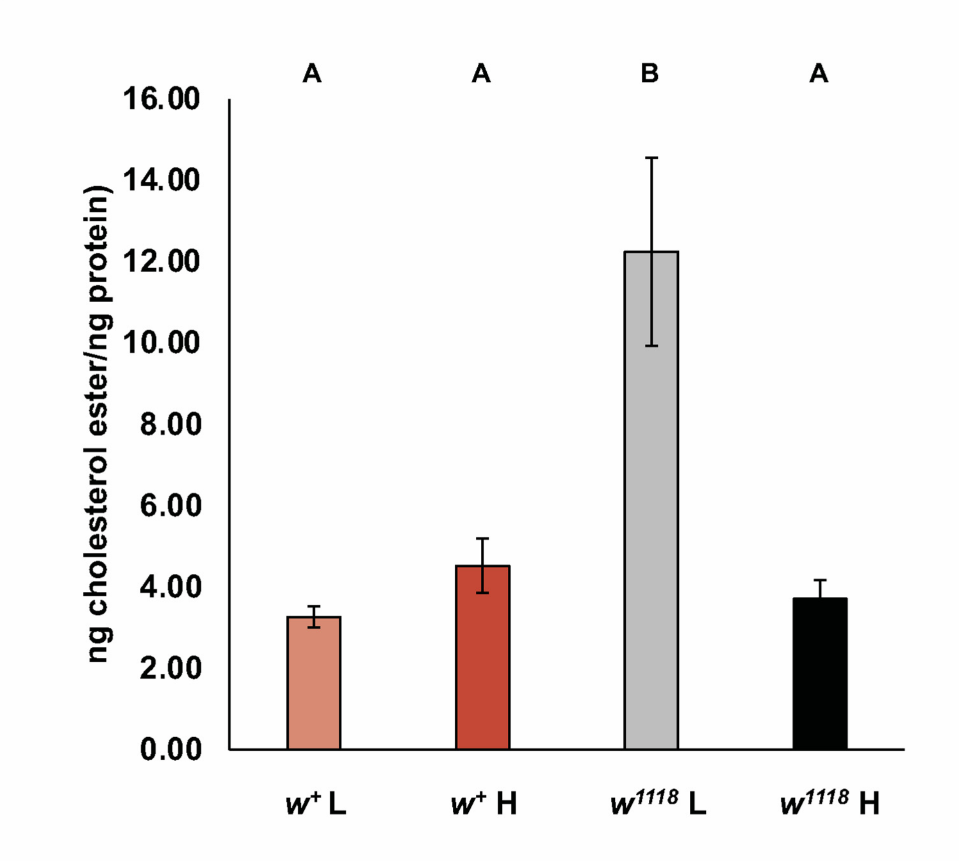

Figure S1. Cholesterol Feeding Differentially Affects Wildtype and *w*[1118] Total Cholesterol Ester Levels. Canton-S (*w*+) and *w*[1118] flies were reared on a low or high (0.1 mg/mL added) cholesterol containing food. *w*[1118] mutants raised on the low cholesterol diet have greatly increased cholesterol esters compared to wildtype controls and *w*[1118] mutants raised on the high cholesterol diet (Two-way ANOVA, Adj. $R^2 = 0.747$, $F_{3,8} = 11.829$, $p < 0.003$, $n = 3$; Tukey low vs. high cholesterol, $p < 0.018$; Tukey w^+ vs. w^{1118}, $p < 0.010$). Groups that do not share letters above the columns are significantly different from each other according to the Tukey post-hoc test ($p < 0.05$).
